# Reply: Reassessing pre-existing comorbidity patterns in testicular cancer: controversies and future directions

**DOI:** 10.1093/hropen/hoaf080

**Published:** 2025-12-13

**Authors:** Marie Juul Ornstrup, Agnethe Berglund, Mads Agerbæk, Claus Højbjerg Gravholt

**Affiliations:** Department of Endocrinology and Internal Medicine, Aarhus University Hospital, Aarhus, Denmark; Department of Clinical Medicine, Aarhus University, Aarhus, Denmark; Department of Endocrinology and Internal Medicine, Aarhus University Hospital, Aarhus, Denmark; Department of Clinical Genetics, Aarhus University Hospital, Aarhus, Denmark; Department of Oncology, Aarhus University Hospital, Aarhus, Denmark; Department of Endocrinology and Internal Medicine, Aarhus University Hospital, Aarhus, Denmark; Department of Clinical Medicine, Aarhus University, Aarhus, Denmark; Department of Molecular Medicine, Aarhus University Hospital, Aarhus, Denmark

Dear Editor,

We thank Chen *et al.* (2025) for their thoughtful comments on our article investigating pre-existing morbidity patterns in men with testicular cancer (TC) using the prospective nationwide Danish Testicular Cancer (DATECA) database (Ornstrup *et al.*, 2025). We appreciate the opportunity to clarify methodological considerations and to elaborate on the strengths and limitations of our study. Below, we address the three main concerns.

First, Chen *et al.* (2025) raise concerns that the control cohort may include men with undiagnosed TC. We acknowledge that any register-based design relies on the accuracy and completeness of national registries; however, several points mitigate the risk of clinically relevant misclassification.

Controls were required to be free of TC diagnoses in *all* relevant national registries: (i) the DATECA database, (ii) the Danish National Patient Registry, and (iii) the National Cancer Registry. We would argue that the exclusion of the 49 controls with a registration of TC detected in *any* of these registries illustrates the effectiveness, not inadequacy, of the multi-registry cross-checking procedure.

Undiagnosed TC is relatively rare, particularly in the universal healthcare system in Denmark with free and easy access to medical evaluation. However, we cannot exclude the risk of having included males with possible undiagnosed TC in the control cohort. Systematic clinical screening (e.g. scrotal ultrasound, serum tumor markers, as suggested) of the entire cohort would indeed minimize theoretical contamination, but would not be feasible in a nationwide epidemiological study of >20 000 individuals, and lies outside the scope of epidemiologic research. Importantly, the likelihood of substantial contamination is low, or non-existent, and any such effect would bias results towards the null, meaning our reported excess morbidity is, if anything, conservative. The lack of clinical data in our epidemiological study is explicitly acknowledged as a limitation within the Discussion section of our article (Ornstrup *et al.*, 2025).

We certainly agree that lifestyle factors, occupational exposures, and environmental risk factors (e.g. perfluoroalkyl and polyfluoroalkyl substances (PFAS)) may contribute to TC risk, as well as to morbidity in other organ systems in general. Below is our response to Chen *et al.*’s (2025) concerns.

A nationwide matched cohort with epidemiological data allows robust comparison of hospital contacts and prescription patterns between cases and controls. Matching 1:10 (case:control) within a universal healthcare system reduces—but does not eliminate—behavioral and environmental confounding. Information on e.g. occupation, smoking habits, and exposure to endocrine disruptors, is simply not available in the national health registries, and therefore cannot be incorporated in studies relying on data from these registries.

The excess morbidity reported was broad, early-onset, and highly consistent across multiple organ systems up to at least 10 years preceding the TC diagnosis, as illustrated in Table 3 of our article (Ornstrup *et al.*, 2025). We believe that the temporal stability and broad disease associations suggest that a single lifestyle or environmental exposure is unlikely to fully explain the pattern.

We explicitly refrain from attributing excess morbidity to specific pathophysiological mechanisms; instead, we emphasize the need to explore potential shared etiological factors—precisely the point raised by Chen *et al.* (2025).

Finally, Chen *et al.* (2025) suggest that excluding only 90 days of health contacts prior to diagnosis may allow TC-related symptoms to be misclassified as pre-existing morbidity. We appreciate this concern and offer the following clarification.

We deliberately tested the robustness of our findings by examining morbidity five and ten years prior to diagnosis, not only the 90-day window. As shown in Table 3 of our article (Ornstrup *et al.*, 2025), the morbidity pattern remained remarkably stable. This strongly suggests that excess morbidity is not driven by symptoms of imminent TC or metastases.

We sincerely thank Chen *et al.* (2025) for their constructive engagement. Their comments highlight important methodological considerations relevant to all epidemiological studies. While these limitations are acknowledged, our conclusion remains unchanged: that men with TC exhibit a substantially increased burden of pre-existing morbidity across multiple health domains, extending beyond the testicular dysgenesis syndrome hypothesis. With that said, we surely agree that future research integrating detailed lifestyle, environmental, and occupational exposure data, where feasible, will be crucial for elucidating the potential underlying mechanisms.
